# Resolving Nonlinear Recombination Dynamics in Semiconductors via Ultrafast Excitation Correlation Spectroscopy: Photoluminescence versus Photocurrent Detection

**DOI:** 10.1021/acs.jpcc.3c04755

**Published:** 2023-08-08

**Authors:** Esteban Rojas-Gatjens, Kaila M. Yallum, Yangwei Shi, Yulong Zheng, Tyler Bills, Carlo A. R. Perini, Juan-Pablo Correa-Baena, David S. Ginger, Natalie Banerji, Carlos Silva-Acuña

**Affiliations:** † School of Chemistry and Biochemistry, Georgia Institute of Technology, Atlanta, Georgia 30332, United States; ‡ Department of Chemistry, Biochemistry, and Pharmaceutical Sciences, University of Bern, Freiestrasse 3, Bern CH-3012, Switzerland; § Department of Chemistry, University of Washington, Seattle, Washington 98195, United States; ∥ Molecular Engineering & Sciences Institute, University of Washington, Seattle, Washington 98195, United States; ⊥ School of Materials Science and Engineering, Georgia Institute of Technology, Atlanta, Georgia 30332, United States; # School of Physics, Georgia Institute of Technology, Atlanta, Georgia 30332, United States

## Abstract

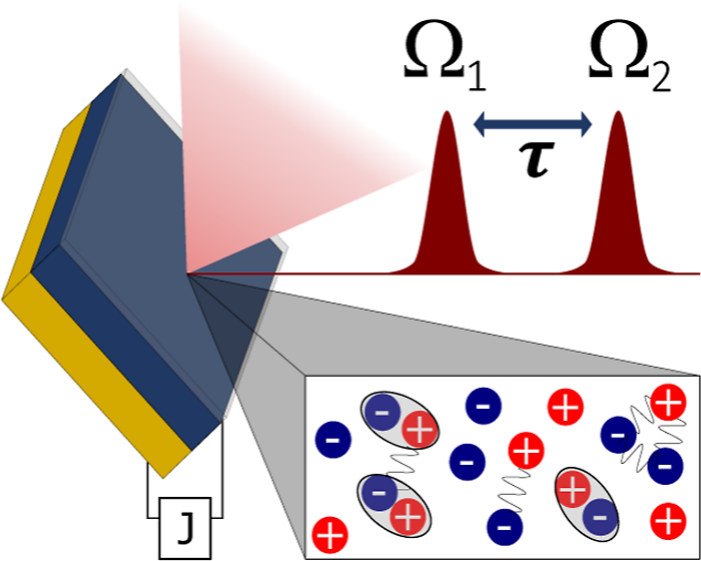

We explore the application of excitation correlation spectroscopy to detect nonlinear photophysical dynamics in two distinct semiconductor classes through time-integrated photoluminescence and photocurrent measurements. In this experiment, two variably delayed femtosecond pulses excite the semiconductor, and the time-integrated photoluminescence or photocurrent component arising from the nonlinear dynamics of the populations induced by each pulse is measured as a function of inter-pulse delay by phase-sensitive detection with a lock-in amplifier. We focus on two limiting materials systems with contrasting optical properties: a prototypical lead-halide perovskite (LHP) solar cell, in which primary photoexcitations are charge photocarriers, and a single-component organic-semiconductor diode, which features Frenkel excitons as primary photoexcitations. The photoexcitation dynamics perceived by the two detection schemes in these contrasting systems are distinct. Nonlinear-dynamic contributions in the photoluminescence detection scheme arise from contributions to radiative recombination in both materials systems, while photocurrent arises directly in the LHP but indirectly following exciton dissociation in the organic system. Consequently, the basic photophysics of the two systems are reflected differently when comparing measurements with the two detection schemes. Our results indicate that photoluminescence detection in the LHP system provides valuable information about trap-assisted and Auger recombination processes, but that these processes are convoluted in a nontrivial way in the photocurrent response and are therefore difficult to differentiate. In contrast, the organic–semiconductor system exhibits more directly correlated responses in the nonlinear photoluminescence and photocurrent measurements, as charge carriers are secondary excitations only generated through exciton dissociation processes. We propose that bimolecular annihilation pathways mainly contribute to the generation of charge carriers in single-component organic semiconductor devices. Overall, our work highlights the utility of excitation correlation spectroscopy in modern semiconductor materials research, particularly in the analysis of nonlinear photophysical processes, which are deterministic for their electronic and optical properties.

## Introduction

Probing photoexcitation dynamics is a cornerstone of material characterization in optoelectronics. Photoexcitations may undergo radiative recombination, defect trapping/detrapping, and high-order processes such as bimolecular and Auger recombination, among others. These recombination dynamics often give rise to a nonlinear response with respect to photoexcitation density, and in turn, they can be determined by resolving time-dependent populations. Researchers commonly probe the nonlinear response using intensity-dependent (i) steady-state photoluminescence (PL) and photocurrent (PC) experiments, in which a deviation of the signal *S*(*I*) from a linear response, *S*(*I*) ∝ *I*^α^ is observed,^1^ (ii) time-resolved PL,^2,3^ or (iii) transient absorption spectroscopies.^4−6^ However, delimiting the distinct nonlinear regimes can be ambiguous between these techniques and may become complex as the system’s components increase. Originally described by von der Linde and Rosen,^7,8^ excitation correlation (EC) spectroscopy provides the means to characterize the nonlinear response with great sensitivity as it is based on double amplitude modulation and phase-sensitive detection. In addition, it maps the time evolution of nonlinear contributions. In EC spectroscopy, we amplitude-modulate two replica ultrafast pulses at frequencies Ω_1_ and Ω_2_, such that demodulating at a reference frequency |Ω_1_ + Ω_2_| using lock-in detection isolates the nonlinear component. EC spectroscopy has been widely used to characterize the carrier lifetimes of several inorganic semiconductors.^9−14^ Despite its utility, neither the organic nor the lead-halide perovskite (LHP) semiconductor community uses it as a routine technique. Only recently, Srimath Kandada et al. employed it to describe the defect density and energetic depth in CH_3_NH_3_PbBr_3_ thin films and CsPbBr_3_ nanocrystals.^15^ The Moran group presented a variation of EC spectroscopy, utilizing a tunable narrow excitation wavelength to characterize layered perovskite quantum-well structures.^16−18^ We have previously implemented ECPL to describe defect states in mixed-halide, mixed-cation metal-halide perovskites.^19,20^

In this article, we implement EC spectroscopy with both PL and PC detection to characterize the nonlinear response of two photodiodes, an LHP in a solar cell, and an organic semiconductor single-component vertical diode. We describe in detail the interpretation of EC signatures using simplified kinetic recombination models that exemplify the class of nonlinear dynamics in these material systems. First, we discuss the typical photophysical processes that result in ECS signals for the case of LHP. We show that trap-assisted and Auger recombination dominate the nonlinear response of LHP devices in PL detection (ECPL) at low and high fluence, respectively. In PC detection (ECPC), the nonlinear components are due to bimolecular and Auger recombination; however, these contributions cannot be easily distinguished with this detection scheme. We also describe the photophysical scenario of the organic semiconductor diode leading to the ECS signal. In this case, where the primary excitation is a Frenkel exciton and charge carriers are not directly generated, ECPL and ECPC provide complementary information about the population evolution of excitons and charges.

## Results

### Nonlinear Dynamics in LHP

**Figure 1:**
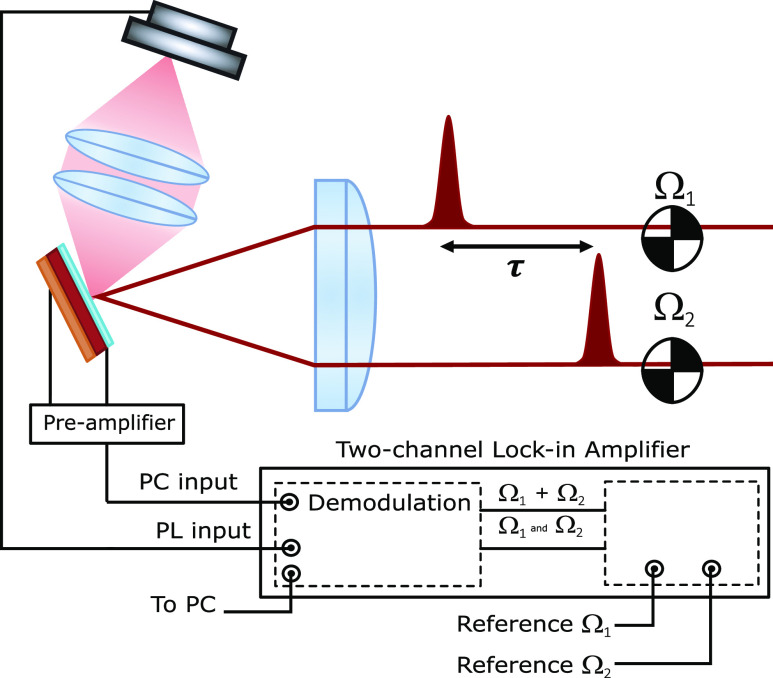
Schematic representation of the EC measurement. The PL signal is measured using a photodiode, and the PC is processed by a current amplifier. Both signals are sent to the lock-in amplifier, which demodulates the input signal at the fundamental of the two amplitude modulation reference waveforms with frequencies Ω_1_ and Ω_2_, and at the sideband Ω_1_ + Ω_2_.

We prepared inverted devices with a mixed-cation mixed-halide perovskite of composition FA_0.83_Cs_0.17_Pb(I_0.85_Br_0.15_)_3_ referred to in the text as Cs17Br15, where FA^+^ refers to the formamidinium cation. Figure S1a, in Supporting Information, shows the linear absorption and PL spectra of a Cs17Br15 film on ITO/MeO-2PACz. Our Supporting Information and previous work^20^ provide further details on the device structure and characterization. Briefly, these films and fabrication procedures yield performance of around 15.90% power conversion efficiency for the best devices. The external quantum efficiency measurement is shown in Figure S2 in the Supporting Information. We emphasize that we perform both ECPL and ECPC on completed device stacks, with typical PL quantum yields in the range of 0.8%. To perform ECPL and ECPC, we excite the sample with a 220 fs pulse with an energy of 2.638 eV and a variable fluence between 1 and 40 μJ cm^–2^. A schematic representation of the EC experiment is represented in Figure 1, and more details about our implementation are described in the appendix.

**Figure 2:**
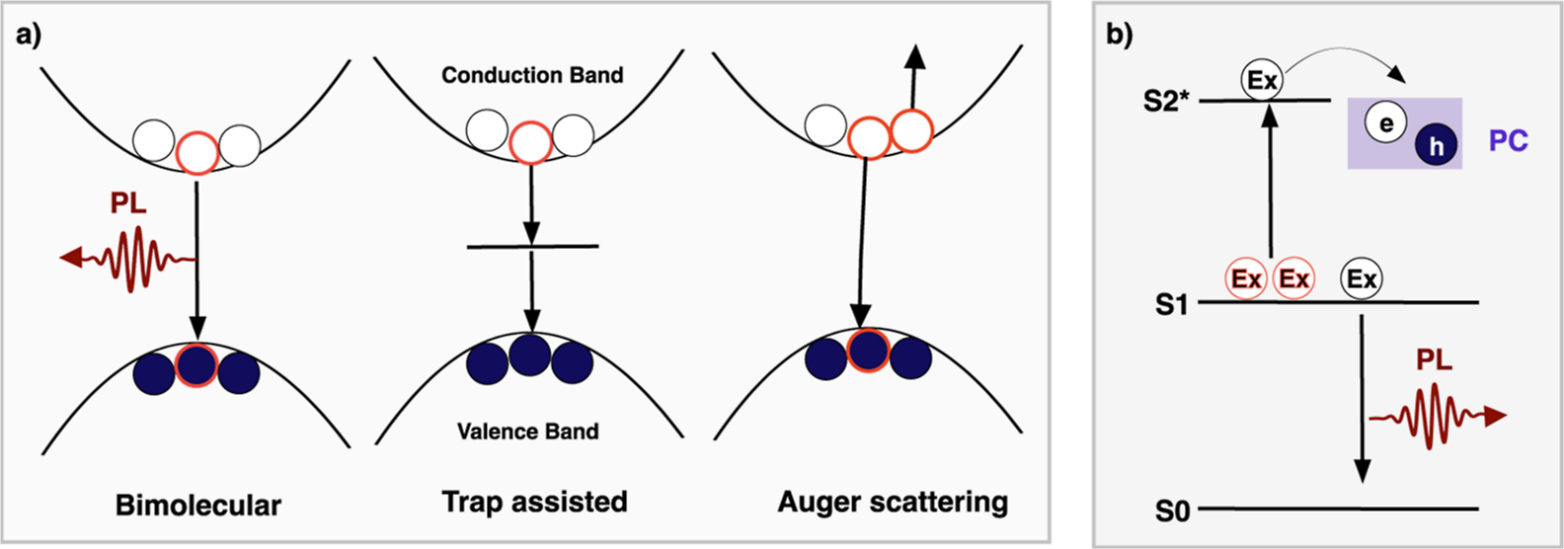
Diagrammatic representation of the recombination kinetic models studied in this work. (a) For FA_0.83_Cs_0.17_Pb(I_0.85_Br_0.15_)_3_, we consider bimolecular, trap-assisted, and Auger scattering as the main recombination pathways. (b) The recombination dynamics of ITIC-4F are represented with a Jablonski diagram, excitons that undergo exciton–exciton bimolecular annihilation acquire enough energy to dissociate.

**Figure 3:**
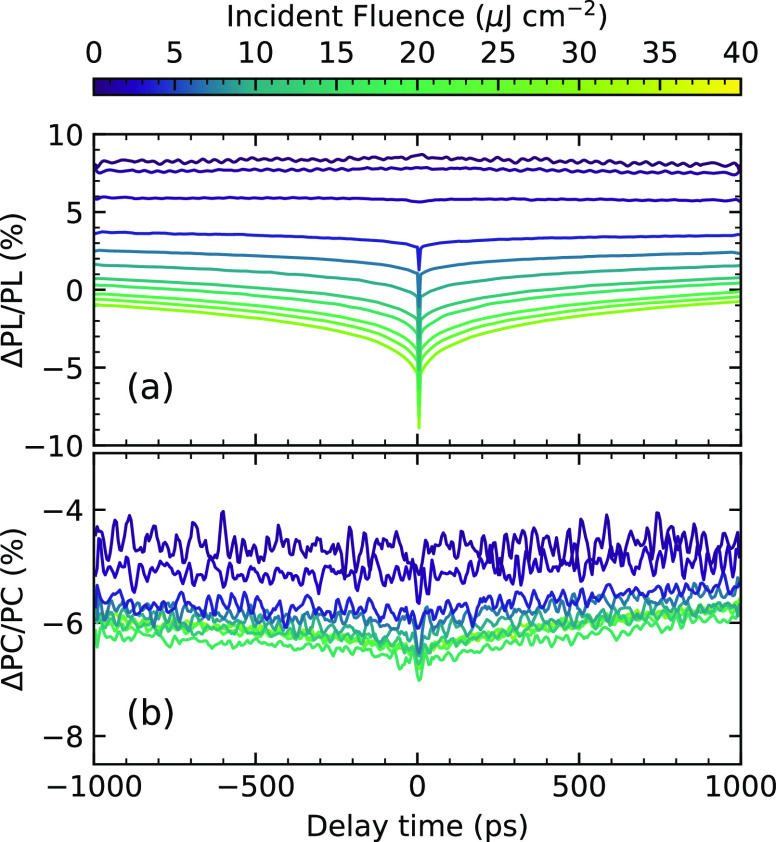
EC spectroscopy measurement of a prototypical Cs17Br15 device. (a) PL detected and (b) PC detected nonlinear response. In both cases, the pump wavelength was 470 nm, and the fluence range is indicated in the false color axis.

Because metal-halide hybrid perovskites are direct bandgap semiconductors, their recombination kinetics involve photocarriers undergoing second-order (bimolecular) radiative recombination of electrons and holes, pseudo-first-order radiative recombination of photogenerated minority carriers with the majority carriers, first-order deep-trap assisted non-radiative recombination, and third-order Auger recombination .^21−23^ These terms are well described by eqs 1–3, where *B* is the bimolecular rate constant, γ_t_ is the carrier trapping rate constant, γ_r_ is the trap recombination rate constant, and γ_Auger_ is the Auger recombination rate constant. Additionally, *N*_t_ and *n*_t_ correspond to empty and occupied trap sites, respectively. The recombination kinetics are diagrammatically represented in Figure 2a. The generation of electrons and holes is assumed to be direct, then their generation populations are considered as initial conditions when solving the differential equations. Note that we do not take into account non-geminate association and dissociation of excitons explicitly since it does not add a distinct recombination order, and additionally, excitons are neither generated nor stable at room temperature.^24^
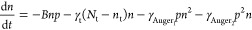
1
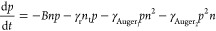
2
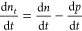
3A distinct model assuming shallow donors has been used to describe CH_3_NH_3_PbBr_3_,^15^ where shallow traps dope the semiconductor. Based on this model, we expect to observe positive subnanosecond dynamics due to fast trapping in shallow traps, accompanied by an increase in the ECPL response as the excitation fluence increases. However, this model does not apply to the Cs17Br15 devices in our study, as discussed below. The ECPL and ECPC measurements are shown in Figure 3a,b, respectively. The time traces show the percentage of the nonlinear signal recovery as we scan the delay between the two pulses; the symmetry between the negative and positive delays indicates that the pulses had a comparable intensity. For the ECPL, at low fluence, we observe a nonlinear response with slow dynamics. The magnitude of the nonlinear response decreases as the fluence increases until it changes sign at the highest fluences. We rule out the shallow donor model as these experimental signatures do not match the model’s prediction that Srimath Kandada et al. described in ref (15). The ECPL signal in Figure 3a shows two distinct regimes: at low fluence, a slow positive trace, and at high fluence, a fast negative nonlinearity. In contrast, the ECPC response, shown in Figure 3b, shows only negative contributions, with no change in the sign of the signal as the fluence increases. To rationalize the information provided by each technique, we interpret the EC measurements of LHPs in terms of the recombination model. Equations 1–3 do not have an analytical solution. However, by making a series of assumptions described below, we can understand the contributions of the specific processes to the nonlinear PL and PC.

### Trap-Assisted Recombination

We follow the assumptions made by previous works on trap-assisted recombination.^9,25^ Specifically, we assume that we are working at a low excitation density, such that Auger recombination does not dominate and can be neglected. Additionally, in materials with low PL quantum yield, as is the case for LHPs, nonradiative trap-assisted pathways typically dominate the carrier recombination such that *Bnp* ≪ γ_t_*N*_t_*n*. Consequently, there is an approximate solution for the electron and hole densities, which reads as
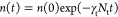
4
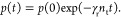
5

We will first discuss the case of ECPL. The detected PL is defined in eq 6. The temporal function describing carriers generated by each pulse is assumed to be a delta function. We then split the integral describing the total PL intensity into two terms: one term considering the carriers photoexcited before the second pulse and another one with carriers photoexcited after the second pulse. Here, *n*_1_(*t*) and *p*_1_(*t*) correspond to the evolution of the carriers according to eqs 4 and 5 after the first pulse arrives, the initial conditions are simply the carriers generated by the first pulse, *n*_1_(0) = *p*_1_(0) = *n*_0_. *n*_2_(*t*) and *p*_2_(*t*) correspond to the evolution of the carriers after the second pulse. These carrier densities are described with the same expression but with distinct initial conditions, *n*_2_(0) = *n*_0_ + *n*_1_(τ) and *p*_2_(0) = *n*_0_ + *p*_1_(τ), as we need to consider residual carriers generated by the first pulse.
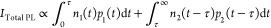
6After integrating eq 6 and subtracting the individual pulse contributions (2*n*_0_^2^), we obtain the nonlinear component of the PL intensity (*I*_NPL_) given in eq 7. Note that the nonlinear term has a positive value, as expected since trap-filling results in a reduction of non-radiative decay pathways. We note that under these assumptions, the *I*_NPL_ follows the same decay as conventional time-resolved experiments. Experimentally, in the ECPL measurements at low fluence shown in Figure 3a, we observe a slow decay rate, which is not entirely captured in the time window of the experiment. This is consistent with eq 7, as the typical values for carriers’ lifetimes in LHPs are between the nanosecond and microsecond ranges. The Supporting Information shows the time-resolved PL experiments in Figure S3.
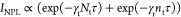
7

We performed a similar analysis for the case of PC detection. The signal measured is defined by eq 8. We ignore the spatial distribution of the carriers and the extraction of carriers for the sake of simplicity. These assumptions affect the magnitude of the nonlinear signal. We interpret the nonlinear PC arising from carrier interactions. The time-resolved PL (see Figure S3) indicates that the recombination kinetics in open-circuit and short-circuit conditions are very similar. Therefore, we justify using the same photophysical scenarios to interpret ECPL and ECPC. It is worth remembering that in ECPC, the time resolution arises from the delay between the pulses instead of from the carrier device extraction. Consequently, we only need the device charge extraction to be faster than the modulation frequency, which is the case by several orders of magnitude.
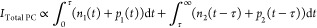
8Under the assumption that trap-assisted recombination dominates at low fluences, the integrands correspond to the monoexponential decay in eqs 4 and 5. This is a linear function with the excitation density; therefore, the nonlinear PC is zero. Trap-assisted recombination does not result in a nonlinear PC response, making ECPC insensitive to traps. However, in the experimental ECPC measurements in Figure 3b, the nonlinear component at low fluence is not zero and has a negative value. Therefore, as discussed below, higher-order processes such as bimolecular recombination and Auger recombination must be responsible for the observed nonlinear PC.

### Bimolecular Recombination

We now consider the case where bimolecular recombination is the dominant recombination pathway. In the Supporting Information, we show that if *Bnp* ≫ γ_t_*N*_t_*n*, then the ECPL response is zero. This approach of ignoring completely the monomolecular recombination does not give an expression for ECPC as the integrals diverge. To attain an approximate analytical expression for the ECPC response, we assume that both bimolecular recombination, *B*, and monomolecular trapping, γ = γ_t_(*N*_t_ – *n*_t_), are present. Also, we assume that holes and electrons have similar trapping rates such that *n* ≈ *p*. This scenario is described in eq 9, and the corresponding solution for the population evolution is shown in eq 10.
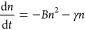
9
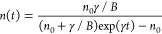
10

After integrating eq 8 and subtracting the individual pulse contribution (4*n*_0_), we obtain an expression (eq 11) that describes the nonlinear PC, *I*_NPC_, where α = *n*_0_*B*/γ. Note that in the limiting cases where there is no bimolecular recombination (*B* = 0), the nonlinear contribution to PC is zero, and at a long delay (τ) between pulses, the expression also goes to zero as the pulses do not overlap in time. Consequently, we can conclude that the nonlinear PC arises from bimolecular recombination and not from carrier trapping, but the time evolution follows the carrier trapping dynamics.
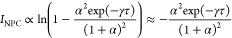
11According to eq 11, the nonlinear PC must have a negative sign, which is congruent with the experimental results shown in Figure 3b. The ECPC measurements, similarly to the ECPL, show slow time dynamics, which is expected as they both follow the time evolution of the carrier population. Additionally, note that in this scenario, the ratio between the bimolecular recombination and the carrier trapping rates dictates the magnitude of the nonlinear PC component.

We have neglected the spatial aspect of the carrier dynamics, which is relevant as we excite the sample in a small area of the sample. Carrier dynamics simulations considering carrier diffusion, carrier trapping, and bimolecular recombination have been carried out by Zhou et al.^18^ for perovskite quantum wells. They observe negative decaying nonlinear PC at longer times, which is congruent with the slow traces shown in Figure 3b. So far, we have rationalized the slow dynamics and the sign of the ECPL and ECPC response at low fluences. Experimentally, as we increase the fluence, we observe a change in sign in the ECPL signal (Figure 2a), while the ECPC response increases in magnitude but remains negative. As the fluence increases, Auger recombination becomes more significant and dominates the recombination kinetics. We will now rationalize the effect of Auger recombination in the nonlinear PL and PC.

### Auger Recombination

We next consider the scenario in which the carrier recombination is dominated by Auger scattering, a third-order process that occurs at high carrier density. Again, assuming that *p* ≈ *n* holds in the high-fluence regime, we describe the kinetics using the rate eq 12. The solution of this equation is presented in eq 13. Here, γ corresponds to the monomolecular recombination rate constant, and *A* corresponds to the Auger recombination rate constant.
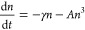
12
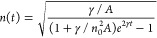
13

In this particular case, the expressions for the nonlinear PL and PC are more complex to evaluate than in the previous scenarios. In the Supporting Information, we show that both the PL and PC exhibit negative nonlinear components due to Auger recombination. This negative contribution explains the fluence-dependent features that we observe for both ECPL and ECPC. In ECPL, when we transition from a trap-dominated recombination scenario with a positive nonlinear component to an Auger-recombination-dominated scenario with a negative nonlinear component, a change of sign is expected. This transition is not expected in ECPC, as both recombination processes (bimolecular and Auger recombination) that result in nonlinear signals lead to negative nonlinear components, making it difficult to distinguish between the two scenarios. Additionally, we note that the ECPL experiments, Figure 3a, show subnanosecond dynamics at the highest fluence (i.e., the sharp decay around zero time delay). We assign these fast features to a large population of carriers recombining through Auger pathways at early times.

### Summary of Nonlinear Dynamics in LHP

In summary, we have described the nonlinear responses caused by the distinct photophysical processes for both PL and PC. We highlight the possibility of distinguishing between trap-assisted and Auger-dominated recombination regimes employing ECPL, as the contributions to the nonlinear response have opposite signs. The excitation density at which the ECPL signal changes sign indicates a change in the dominant process, which is related to the number of traps, meaning that ECPL is a good technique to characterize trap densities in LHPs. On the other hand, in ECPC, the nonlinear signal arising from bimolecular recombination and Auger recombination has the same sign (negative); monomolecular recombination by itself does not result in a nonlinear signal. Therefore, ECPC does not provide as rich information about trap density as ECPL since the signatures are convoluted and difficult to isolate. Recent work by Zhou et al.^16,18^ explores the implementation of a similar experimental setup to characterize carrier diffusion; this idea is not expanded in this work as large time scales (tens of nanoseconds), that exceed our implementation capabilities, are needed.

### Nonlinear Dynamics in Organic Semiconductors

In organic semiconductors, the primary photoexcitation is a neutral exciton. Charge carriers are generated after the dissociation of the exciton, which can occur through several mechanisms in the neat semiconductor. One such mechanism is the formation of an intermediate charge transfer state prior to charge separation,^26^ although the precise mechanism for this process is not clear and is certainly not trivial. Another mechanism for exciton dissociation is to overcome the exciton binding energy by promoting the exciton to a higher-energy excited state, *S*_n_*. This can be achieved by coherent two-step photo-excitation pathways using femtosecond-pulse excitation, described as an excitation from *S*_0_ to *S*_1_ and subsequently from *S*_1_ to *S*_n_*. The process generates a high-energy state prone to relaxation due to charged excitations (polarons) and triplet-excitons.^4,27,28^ Alternatively, the *S*_n_* state can be reached through energy transfer between excitons in a process known as exciton–exciton annihilation,^4,27−29^ this is the mechanism proposed for this work, as discussed below and represented with a Jablonski diagram in Figure 2b.

The two detection methods used in EC spectroscopy presented here are each sensitive to different excited-state species produced optically in organic semiconductors. While the charge carriers were both the emissive species and the PC-detected species in LHPs, excitons and charges can be observed individually in the neat organic devices studied here. Excitons correspond to the detected emissive species (Figure S1b in Supporting Information), and charges arising from subsequent exciton dissociation result in the detected PC. The ECPL experiment then provides insights into the processes leading to exciton recombination, while the ECPC provides information on those resulting in charge-carrier generation and recombination. In this work, we assess the photophysical processes occurring in neat ITIC-4F. We prepared a single-component device with an architecture ITO/ZnO/ITIC-4F/MoO_3_/Ag and measured both ECPL and ECPC. The absorption and PL emission spectra of ITIC-4F are shown in Figure S1b in the Supporting Information. Further details about the device preparation are presented in the Supporting Information. The pump pulse used for these EC spectroscopy experiments has an energy of 1.823 eV.

### Nonlinear PL

Consider the simple model in eq 14, where the monomolecular rate γ incorporates all monomolecular processes, including radiative and non-radiative relaxation pathways, and β is the bimolecular exciton annihilation rate. This model is mathematically equivalent to eq 9 discussed for LHPs. As shown above, eq 14 can be solved analytically and leads to the expression for the nonlinear PL shown in eq 15. We define α = *n*_0_β/γ equivalently.
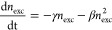
14
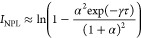
15

**Figure 4:**
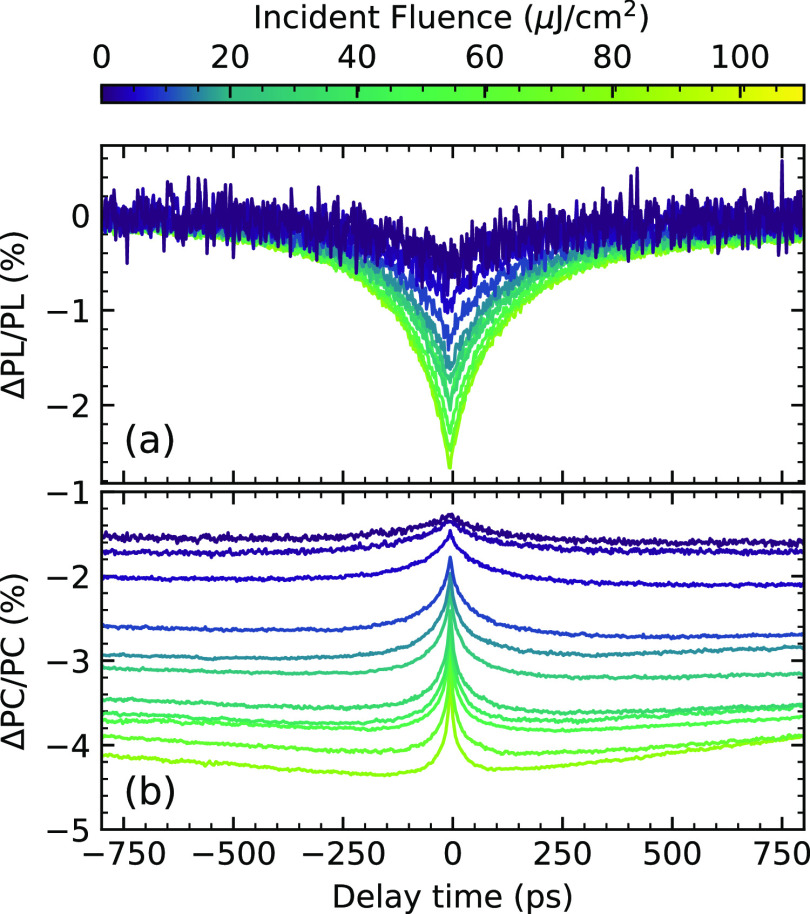
EC spectroscopy measurement of an ITIC-4F device. (a) PL detected and (b) PC detected nonlinear photocarrier dynamics. The pump wavelength was 680 nm, and the fluence range is represented by the false color axis.

Figure 4a shows the experimental ECPL response for a range of fluences between 1 and 100 μJ cm^–2^. It can be observed that the magnitude of the nonlinear response increases with fluence, as expected since exciton–exciton annihilation becomes more significant as the exciton density increases. By fitting the measured traces to eq 15, we extracted a value of monomolecular recombination rate of (6.3 ± 0.5) × 10^9^ s^–1^. We extracted the bimolecular rate using the experimental setup discussed by Riley et al. in ref (3) and obtained a value of (1.0 ± 0.2) × 10^–9^ cm^3^ s^–1^ similar to those reported previously in the literature.^3^ A summary of the analysis and fitted data are shown in Supporting Information, in Figures S5 and S6. In the experimental configuration of ref (3), the material is excited with a single pulse whose amplitude is modulated by a mechanical chopper. The bimolecular exciton–exciton rate was obtained after analyzing the fluence dependence of PL intensity.

In this section, using a simple model including monomolecular and exciton–exciton annihilation, we show that ECPL follows the excitons population time evolution through the nonlinear PL generated in organic semiconducting materials. More complex scenarios involving coexisting excitonic species have been analyzed previously.^11,13^ In those cases, the additional exciton dynamics afflict the spectral integrated response, as the one measured in our work. Spectrally resolving the signal is necessary to distinguish nonlinear dynamics from distinct emissive species with similar emission energy.

### Nonlinear PC

To interpret the PC response in organic semiconductors, we will focus on a simple model for charge carrier population dynamics described by eq 16. In this model, the photocarriers are generated through the function *G*(*t*), which depends on the photophysical process that results in charges. We acknowledge that the generation of photocarriers in neat organic semiconductors and a complete description of their dynamics is a complex problem and that multiple techniques are needed to provide a robust physical picture. However, in this work, we focus on the contributions that ECS can bring to the field, and thus we provide our hypothesis of the photophysical scenario in this materials class.

**Figure 5:**
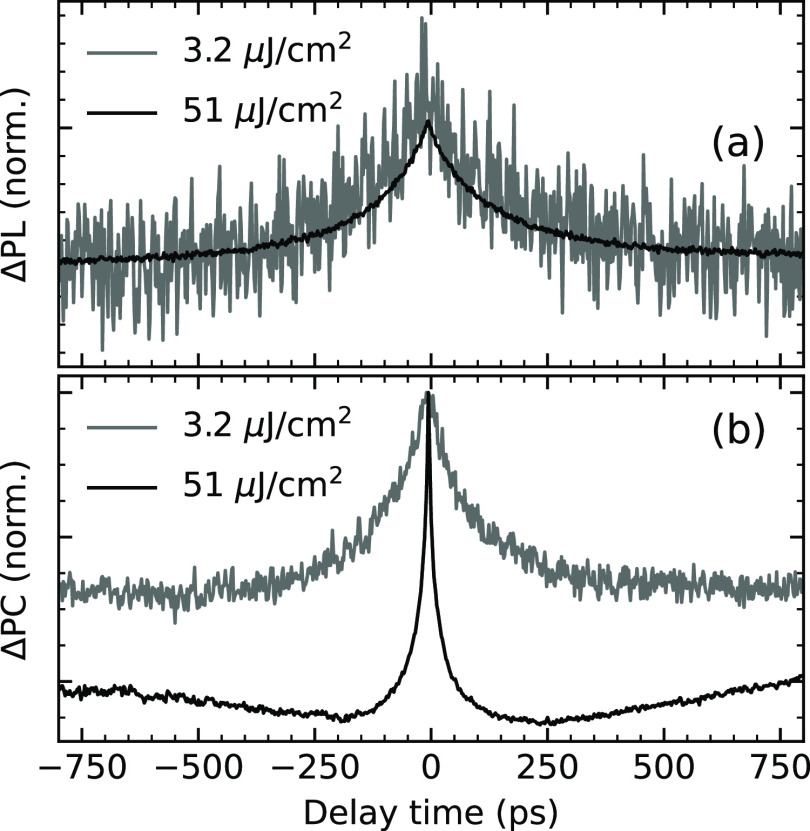
Normalized nonlinear response. (a) PL detected and (b) PC detected signal.

In our simplified model, we assume that the dynamics of electron and hole carriers are comparable, which is very likely as the system is not doped. Additionally, γ_D_ is the monomolecular decay rate of the carriers, and γ_B_ is the non-geminate recombination rate. Note that eq 16 is similar to eqs 9 and 14. From this, we can deduce that the nonlinear PC (ECPC) will have a negative sign due to the non-geminate recombination experienced by the carriers and that the time trace will follow the carrier’s time evolution. In this case, however, we need to consider a time-dependent generation term. We cannot assume it to be a delta function. This makes an analytical solution challenging.
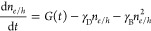
16Experimentally, in the ECPC response, shown in Figure 4b and 5b, we observe a “rising” feature. We interpret this feature as the generation time of charge carriers. The nonlinear interactions depend on the delay time between the pulses since the carriers’ populations evolve with time. The nonlinear PC signal is negative, and the amplitude at *t*_0_ would ideally be zero. However, we believe the offset around *t*_0_ is related to long-lived carriers, which could be fluence-dependent. Note that as the incident fluence increases, the generation rate increases as well. Based on this experimental observation, we discuss possible generation mechanisms below.

Consider the case of a charge carrier being generated through a two-step excitation, as has been proposed for several polymeric materials .^4,27,28,30^ For this case, since the photocarriers are directly pumped, the generation function is *G*(*t*) ∝ δ(*t*). We can discard this as the dominant mechanism, as we expect it to manifest in the ECPC as a maximum in the absolute signal when the two pulses temporally overlap. Instead, experimentally, we observe a minimum. A similar experiment as the one presented here probed the two-step excitation pathway and observed that the response followed the exciton decay dynamics.^30^ At high fluences, they observed a “rise” time interpreted as a fast relaxation from a hot vibrational state *S*_1_* to *S*_1_, followed by a subsequent excitation from *S*_1_ to *S*_*n*_. The rise time that we observed is too long to correspond to any relaxation time, which is usually in the sub-picosecond time scale.^30^

Other possible mechanisms are charge generation after bimolecular exciton–exciton annihilation^27^ and a recently proposed monomolecular exciton dissociation.^31^ In both cases, the generation rates will depend on the population of the excitons. In the bimolecular exciton–exciton annihilation pathway, the generation function would be proportional to the square of the exciton population, *G*(*t*) ∝ *n*(*t*)^2^. While for monomolecular exciton dissociation, the generation rate is proportional to the exciton population, *G*(*t*) ∝ *n*(*t*). Notice in Figure 4b, further exemplified in Figure 5b, that the “rise” of the nonlinear response is dependent on the excitation density. Figure S7 shows a multi-exponential fit to further quantify the differences, although we do not assign specific physical meaning to the values extracted. Since the excitons population evolution *n*(*t*) depends on the excitation density, both mechanisms show the same trend to become faster as the fluence increases. However, since we observed experimentally a more dramatic effect in the PC detection scheme than in the PL scheme, shown in Figure 5, we hypothesize that the dominant generation mechanism is bimolecular annihilation of the exciton population.

Furthermore, the charge lifetime observed in ECPC (Figure 4b) is considerably longer than the exciton lifetime (Figure 4a). We cannot quantify it as it is outside of the instrument’s temporal window. As the fluence increases, we observe that the decay of the charge becomes more significant, indicating that the bimolecular recombination of carriers becomes more important, as expected from the simple model presented above. Finally, we acknowledge that there are other causes of nonlinear PC that were not discussed in this work, e.g., the current limitation due to the external resistance series.^32^ We tried to minimize these effects by performing the measurements at a low fluence range.

### Summary of Nonlinear Dynamics in Organic Semiconductors

We have shown how ECPL can be used to extract photophysical parameters like monomolecular and bimolecular decay rate constants. In the ECPC experiments, we interpret the “rising” features around zero time delay as the time-dependent generation of charge carriers, which becomes faster with increasing fluence. We suggest that charge carriers are generated through bimolecular annihilation pathways. Since the fluence dependence of the generation time might originate from a second-order charge generation process, *G*(*t*) ∝ *n*^2^. Other mechanisms could also be involved; however, due to the strong PL nonlinearity observed, we expect bimolecular exciton–exciton annihilation to be the dominant pathway toward charge generation. To further clarify the complete photophysical scenario, systematic experiments using complementary techniques (e.g., transient absorption and time-resolved PL spectroscopies) are needed and recommended as future endeavors.

## Discussion

In this work, we presented two scenarios in which the nonlinear PL and PC detection schemes provide distinct and complementary information. In the case of LHPs, the same excited-state species contribute to both the nonlinear PL and PC. Therefore, via ECPL and ECPC, we follow the population of the same species; however, this species leads to distinct nonlinear responses in each of the physical observables. This offers the possibility to selectively study photophysical processes experienced by the excited species based on the detection scheme. For example, we showed that while the ECPL shows a response due to trap-assisted recombination, the ECPC is insensitive to traps themselves. The magnitude of ECPC, instead, is given by bimolecular recombination, a process to which ECPL is insensitive. As mentioned above, Zhou et al.^18^ have taken advantage of this to characterize carrier diffusion in layered perovskites using PC detection.

For the case of organic semiconductors, we probe exclusively the excitons that recombine radiatively or those that dissociate and generate charge carriers with each detection scheme, PL and PC, respectively. In that sense, the work presented here adds to the existing toolbox of ultrafast PL and PC techniques,^33,34^ with the additional feature that the magnitude of the response can be used to describe the rates responsible for the nonlinearities. In this material, both ECPL and ECPC have negative nonlinear responses which are directly related to the exciton–exciton bimolecular annihilation rate and the charge carrier bimolecular recombination, respectively. As the excitonic scenarios become complex, the magnitude of the nonlinear signal provides insight into nonlinear processes occurring on the ultrafast scale hidden to steady-state measurements or that appear convoluted in time-resolved techniques.

It is worthwhile to contextualize the ECS experiments with others from the community. Mainly, Kiligaridis^23^ and co-workers present a one-pulse experiment to analyze the recombination dynamics in LHPs. The basis of the experiment is similar to the one presented here, as it relies on analyzing the relative PL quantum yield (rPLQY). In their work, they emphasize the importance of including Auger recombination and trapping models to reproduce the intensity dependence of the relative PL quantum yield. Additionally, as mentioned above, Riley et al.^3^ present a one-pulse experiment for the characterization of organic semiconductors using the same model presented here to interpret the data. Our work expands on their analysis by introducing an additional pulse to isolate the nonlinear component of the response and resolve the population’s time evolution.

As mentioned in the Introduction, recent reports describe a variation of the ECS probe, utilizing a tunable narrow excitation wavelength to characterize layered perovskite quantum-well structures.^16−18^ We note that in their interpretation, there is ambiguity in the distinction between the incoherent and coherent contributions to the nonlinear response. The measured spectra are interpreted as 2D excitation spectra, but we highlight that there is no well-defined phase resolution in the excitation-pulse wave packets, and the measurements are thus purely incoherent, as in the work presented here. This incoherent response arises from the dependence of the physical observable on the intensity of the excitation due to the population evolution (e.g., trap recombination and exciton–exciton recombination), rather than a coherent nonlinear response as in coherent multidimensional spectroscopies.^35−39^ We also note that these 2D measurements that implement phase modulation may also contain incoherent contributions due to nonlinear population dynamics picked up by the phase demodulation detection scheme.^40−42^ We thus underline the difference between the technique presented in this article and 2D coherent excitation. Earlier, ECS-like experiments have been interpreted using Feynman diagrams.^16,17^ We emphasize that this is not precise since Feynman diagrams indicate optical transitions among states and their coherent correlation but do not include the interactions among their populations. This imprecision is addressed in recent literature, recognizing recombination dynamics as the only origin of the measured nonlinearity.^43^ Due to their distinct origin, they provide distinct information. While 2D coherent excitation experiments provide information regarding dephasing rates and coherent correlations between excited states, the ECS experiments provide information uniquely about population mixing. In this work, we expand on the signal-generation mechanisms associated with population mixing. Together with previous examples;^7,8,10−15,19,20^ our work adds another tool to the modern semiconductor community for the characterization of nonlinear photophysical processes.

## Conclusions

We have observed and rationalized the main nonlinear signatures in the PL and PC of LHP and organic semiconductor devices. For the case of LHPs, the ECPL has nonlinear components due to trap-assisted and Auger recombination with opposite behavior, sublinear and supralinear. The fluence dependence of ECPL provides rich information about defect density, ultrafast dynamics, and Auger recombination. Meanwhile, in ECPC, the nonlinear signature originates from bimolecular and Auger recombination, both of which are supralinear processes. In ECPC, the nonlinear contributions are convoluted and difficult to distinguish. In organic semiconductors, we describe ECPL as a sensitive technique for determining exciton-annihilation rates. On the other hand, ECPC represents a valuable tool to study charge generation through photoexcitation. The experimental data suggest that ECPC can follow the population dynamics of free charges, including their generation dynamics. Additionally, from the rise time observed in ECPC, we hypothesize that bimolecular annihilation corresponds to a significant pathway for charge carrier generation.

We expect EC spectroscopy to have an impact, particularly in the field of organic electronics, where it can shine further insight into the physical nature of excited states and the generation mechanisms leading to charge carriers. Besides the case of study of single-component materials, mixed systems with complex fluence-dependent photophysical processes will also benefit from EC spectroscopy. For example, in recent studies of perovskite-sensitized TTA-UC, the intensity dependence of PL shows an interplay of processes with distinct nonlinearities,^1^ which could be better resolved by EC spectroscopy as well as their time-resolved dynamics.

## Supporting Information Available

Details of the experimental setup, signal acquisition, solar cell, and diode used; details on the ECPL and ECPC models; and curve-fitting parameters (PDF)
