# Supplementary material for: Resolving Nonlinear Recombination Dynamics in Semiconductors via Ultrafast Excitation Correlation Spectroscopy: Photoluminescence versus Photocurrent Detection
Source: J Phys Chem C Nanomater Interfaces. 2023 Aug 8;127(32):15969–77. doi: 10.1021/acs.jpcc.3c04755 (PMC10440815; doi:10.1021/acs.jpcc.3c04755)
Supplement: Supplementary file 1 — jp3c04755_si_001.pdf [file jp3c04755_si_001.pdf]

Resolving Nonlinear Recombination Dynamics in Semiconductors via Ultrafast Excitation Correlation Spectroscopy: Photoluminescence versus Photocurrent Detection

Esteban Rojas-Gatjens[1], Kaila Yallum[2], Yangwei Shi[3,4], Yulong Zheng[1], Tyler Bills[1], Carlo Andrea Riccardo Perini[5], Juan-Pablo Correa-Baena[5], David S. Ginger[3], Natalie Banerji[2], Carlos Silva-Acuña[‡1,5,6]

[1] *School of Chemistry and Biochemistry, Georgia Institute of Technology, 901 Atlantic Drive, Atlanta, Georgia 30332, USA.*

[2] *Department of Chemistry, Biochemistry, and Pharmaceutical Sciences, University of Bern, Freiestrasse 3, CH-3012 Bern, Switzerland*

[3] *Department of Chemistry, University of Washington, Seattle, WA 98195, USA.*

[4] *Molecular Engineering & Sciences Institute, University of Washington, Seattle, WA 98195, USA.*

[5] *School of Materials Science and Engineering, Georgia Institute of Technology, North Avenue, Atlanta, GA 30332, United States.*
E-mail: carlos.silva@gatech.edu

[6] *School of Physics, Georgia Institute of Technology, 837 State Street, Atlanta, Georgia 30332, United States.*

‡ *Currently visiting as Honorary Professor, Departamento de Física Aplicada, Centro de Investigación y de Estudios Avanzados del Instituto Politécnico Nacional, 97310 Mérida, Yucatán, México.*

Contents

1 Experimental details: S2

1.1 Excitation Correlation Spectroscopy . . . S2
1.1.1 Signal recovery from lock-in amplifier . . . S2
1.1.2 Trap-assisted recombination . . . S2
1.1.3 Bimolecular Annihilation . . . S3
1.1.4 ECPC discussion. Perovskite solar cell case . . . S4
1.2 Sample details . . . S4
1.2.1 Perovskite solar cell devices . . . S4
1.2.2 Organic single component device . . . S4

2 ECS supporting data and models S7

2.1 Lineal photoluminescence and photocurrent . . . S7
2.2 Analytical models . . . S7
2.2.1 Trap-assisted recombination: . . . S7
2.2.2 Bimolecular recombination: . . . S8
2.2.3 Auger recombination: . . . S8
2.3 Fitting procedures . . . S9
2.3.1 Quasi-Steady . . . S9
2.3.2 ECPL . . . S9
2.3.3 ECPC . . . S9

1 Experimental details:

1.1 Excitation Correlation Spectroscopy

In our implementation, 1030 nm, ∼220 fs pulses are generated in an ultrafast laser system at a 100 kHz repetition rate (PHAROS Model PH1-20-0200-02-10, Light Conversion). A portion of the laser beam is sent into a commercial optical parametric amplifier (ORPHEUS, Light Conversion). The pulse trains are then split 50/50 by a beam splitter cube, where one of the beams is directed to a motorized linear stage (LTS300, Thorlabs), allowing for control of the delay between the two pulses. Each pulse is modulated with a chopper at the frequencies of 373 and 199 Hz, respectively, and the pulses are then focused onto the sample with a 100 mm focal length lens. The total integrated response and the nonlinear component are obtained simultaneously by demodulating both the fundamental and the sum of the modulation frequency. Photoluminescence detection (ECPL): The emitted PL is filtered with a long-pass filter to get rid of the pump, and then it is focused into a photoreceiver (New Focus 2031 PR) connected to a lock-in amplifier (HF2LI, Zurich Instruments). Photocurrent detection (ECPC): The device is connected to a Zurich Instruments HF2TA Current Amplifier used to convert the current output of the sample device to voltage, as well as to supply an external bias to the device. The current amplifier is connected to a lock-in amplifier (HF2LI, Zurich Instruments). The photocurrent measurements presented here were acquired with no external applied bias.

1.1.1 Signal recovery from lock-in amplifier

Additionally, in this appendix, we expand on the experimental details for measuring the nonlinear component utilizing double modulation lock-in detection. The intention is to provide two examples of nonlinear photophysics processes recovered through double demodulation and to bring attention to the fiendish experimental details. We define the generation rate to take into account the repetition rate and $S(t,\omega)$ to be a square wave to mimic the chopper.

$$G(t,\omega) = gS(t,\omega)\sum_{n}^{\infty}\delta(t - nt_{rep}) \tag{S1}$$

Remember that the square wave function that alternates between 0 and 1 is given as:

$$S(t,\omega) = \frac{1}{2} + \frac{2}{\pi}\sum_{n=0}^{\infty}\frac{\sin((2n+1)\omega t + \theta)}{(2n+1)}. \tag{S2}$$

Consider the cases where the reference signal, with which the signal is demodulated, corresponds to a sine function or a square wave. Also, we will ignore the phase as this can be easily set experimentally.

1.1.2 Trap-assisted recombination

We take $\gamma = \gamma_r N_r = \gamma_t n_t$ and both pulses to have the same intensity. Then, using the equations S17 we integrate $\int_0^{t_{rep}} Bn(t)p(t)dt$ which corresponds to the response of the detector. Since t_{rep} is much longer than the carrier lifetime we integrate from zero to infinity instead and obtain the intensity:

$$I(t,\omega_1,\omega_2) \propto \frac{S(t,\omega_1)+S(t,\omega_2)}{2} + S(t,\omega_1)S(t,\omega_2)e^{-\gamma\tau}. \tag{S3}$$

All the constants were grouped with the response of the detector.

We mimic the demodulation of the lock-in amplifier by multiplying the signal by:

$$S_{ref}(t,\omega) = A\sum_{n=0}^{\infty}\frac{\sin((2n+1)\omega t)}{(2n+1)}. \tag{S4}$$

. Then we average over a long time such that oscillating components vanish. Then the intensity recovery for each modulation frequency is:

$$\langle I_{mod}(\omega_1)\rangle_{LI} \propto \frac{A}{2\pi}\sum_{n=0}^{\infty}\frac{1}{(2n+1)^2}\left(1+e^{-\gamma\tau}\right). \tag{S5}$$

$$\langle I_{mod}(\omega_1)\rangle_{LI} = \langle I_{mod}(\omega_2)\rangle_{LI} \propto \frac{A\pi}{16}\left(1+e^{-\gamma\tau}\right). \tag{S6}$$

Note that part of the mixed term is recovered in the single modulation since $\langle S(t,\omega)\rangle = 1/2$. Now, we expand the mixed term to:

$$\begin{aligned} S(t,\omega_1)S(t,\omega_2) = \frac{1}{4} + \frac{1}{\pi}\sum_{i=0}^{2}\sum_{n=0}^{\infty}\frac{\sin((2n+1)\omega_i t)}{(2n+1)} + \\ \frac{4}{\pi^2}\sum_{n,m=0}^{\infty}\frac{\sin((2n+1)\omega_1 t)}{(2n+1)}\frac{\sin((2m+1)\omega_2 t)}{(2m+1)}. \end{aligned} \tag{S7}$$

After we demodulate at the sum frequency $\omega_1+\omega_2$ and average a long time. The only terms that survive come from the last sum, when n and m are the same. Then:

$$\langle S(t,\omega_1)S(t,\omega_2)S_{ref}(t,\omega_1+\omega_2)\rangle = \frac{A}{2\pi^3}\sum_{n=1}^{\infty}\frac{1}{(2n+1)^3} = A\epsilon. \tag{S8}$$

$$\langle I_{mod}(\omega_1+\omega_2)\rangle_{LI} \propto A\epsilon e^{-\gamma\tau}. \tag{S9}$$

1.1.3 Bimolecular Annihilation

We choose the delay between the pulses to be zero for simplicity. From the equation above, the total photoluminescence detected is:

$$I_{Total\,PL} \propto \int_0^{\infty} n(t)dt \propto \ln\left[1 + n_i\frac{\gamma_A}{\gamma_{eff}}\right]. \tag{S10}$$

We define $n_i = g(S(t,\omega_1)+S(t,\omega_2))$. Then, we do a second-order Taylor expansion, and $\alpha = g\gamma_A/\gamma_{eff}$.

$$\approx \alpha(S(t,\omega_1)+S(t,\omega_2)) - \frac{\alpha^2}{2}(S(t,\omega_1)+S(t,\omega_2))^2. \tag{S11}$$

Remember that the square wave is an idempotent function.

$$= \alpha(S(t,\omega_1)+S(t,\omega_2))\left(1-\frac{\alpha}{2}\right) - \alpha^2 S(t,\omega_1)S(t,\omega_2). \tag{S12}$$

Then after demodulating with a square function, we obtained:

$$\langle I_{mod}(\omega_1)\rangle_{LI} = \langle I_{mod}(\omega_2)\rangle_{LI} \propto \frac{A\pi}{8}\alpha\left(1-\alpha\right). \tag{S13}$$

$$\langle I_{mod}(\omega_1+\omega_2)\rangle_{LI} \propto -A\epsilon\alpha^2. \tag{S14}$$

1.1.4 ECPC discussion. Perovskite solar cell case

To ensure collection of the ECPC signal, all current transients should be completed at a speed faster than the lock-in modulation used. The response time of a solar cell under pulsed illumination is computed as:

$$t_R = \sqrt{t_{Drift}^2 + t_{Diffusion}^2 + t_{RC}^2} \tag{S15}$$

Where t_{Drift} is the charge collection time for charges in the depleted region of the junction, $t_{Diffusion}$ is the collection time for charge carriers in the undepleted region, and t_{RC} is the response time induced by the combination of the diode and the circuit. For a perovskite solar cell of about 1 cm^2 area, the intrinsic response times are significantly faster than t_{RC}. The response time measured can therefore be approximated as:

$$t_R = t_{RC} \tag{S16}$$

Where $t_{RC} = 2.2\,RC$, where R is the sum of the diode series and amplifier input resistances, and C is the sum of the solar cell junction and stray capacitances. In our system R $\approx 50\,\Omega$, the input resistance of the amplifier, and C $\approx$ 100 nF, the capacitance of the solar cell junction (assuming 1 nF/mm^2 area capacitance and 1 cm^2 area). Therefore $t_{RC} \approx 10\,\mu s$ (10^5 Hz), and the system response remains orders of magnitude faster than the fastest of the chopper frequencies detected by the system $\Omega_1 + \Omega_2 = 572\,\text{Hz}$.

1.2 Sample details

1.2.1 Perovskite solar cell devices

For the perovskite solar cell devices, we prepared inverted devices with a mixed-cation mixed-halide perovskite of composition $FA_{0.83}Cs_{0.17}Pb(I_{0.85}Br_{0.15})_3$ (denoted as Cs17Br15) and device architecture ITO/MeO-2PACz/Cs17Br15/C60/BCP/Ag. The Patterned ITO glass substrates were thoroughly cleaned by sonicating them in water (with 2 % Micro-90 detergent), deionized water, acetone, isopropanol(IPA) for 10 mins, respectively, followed by plasma cleaning for 5 mins. 1 mmol/L of MeO-2PACz solution was used for spin-coating on top of ITO substrates with 3000 rpm for 30 s, which was then annealed at 100 °C for 10 min. The perovskite with a concentration of 1.2 M (dissolved in DMF:DMSO = 4:1 in volume ratio) layer was spin-coated at 4000 rpm for 60 s. Chlorobenzene (CB) antisolvent was dropped on top when 35 s remained. The perovskite films were annealed at 100 degrees for 30 s and 150 °C for 10 min. After spin-coating the perovskite layer, 30 nm C_{60} and 5 nm bathocuproine (BCP) were thermally evaporated, followed by 100 nm of Ag. We measured the current density-voltage (J-V) curves of the devices using a Keithley 2400 source meter under 1 Sun illumination (AM 1.5G, 100 mW/cm2) in a nitrogen glovebox. The light source was calibrated with a filtered KG3 silicon reference solar cell. The J-V curves were recorded in the range of -0.1-1.2 V with a step of 0.02 V. The solar cell devices were masked with a metal aperture (0.0453 cm2) to define the active area.

1.2.2 Organic single component device

The organic semiconductor devices were prepared using the non-fullerene acceptor large molecule ITIC-4F with an architecture ITO/ZnO/ITIC-4F/MoO_3/Ag. A 10

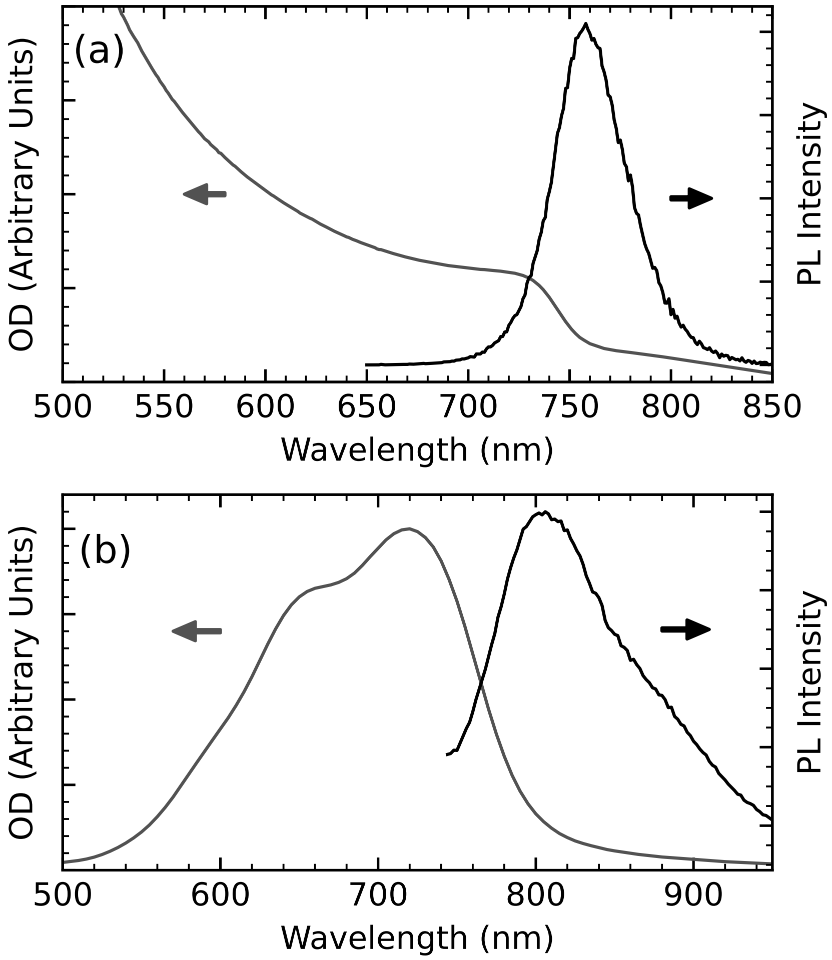

Figure S1: Absorbance and PL for (a) Cs17Br15 half-stack device (ITO/MeO-2PACz/Cs17Br15) and (b) ITIC-4F samples.

nm layer of ZnO was spincoated from 70 μL of 0.1 M ZnO nanoparticles in 2-methoxyethanol at 3000 rpm for 45 seconds. After spincoating, this layer was dried at 120° C for 10 minutes and brought to room temperature on the cooling hotplate. The active layer had a thickness of 85 nm, spincoated from 60 μL of an 8 mg/mL ITIC-4F solution in chloroform at 800 rpm for 55 seconds. MoO_3 was deposited by evaporation at a rate of 0.1 Angstrom/s with an ultimate thickness of 5 nm. A 100 nm layer of Ag was deposited by evaporation at a rate of 1 Angstrom/s.

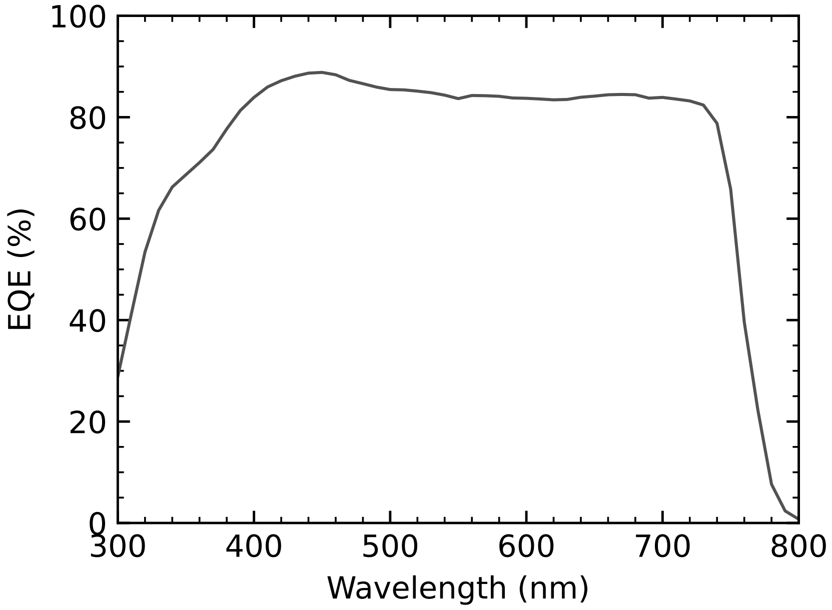

Figure S2: External quantum efficiency (EQE) of Cs17Br15 full device.

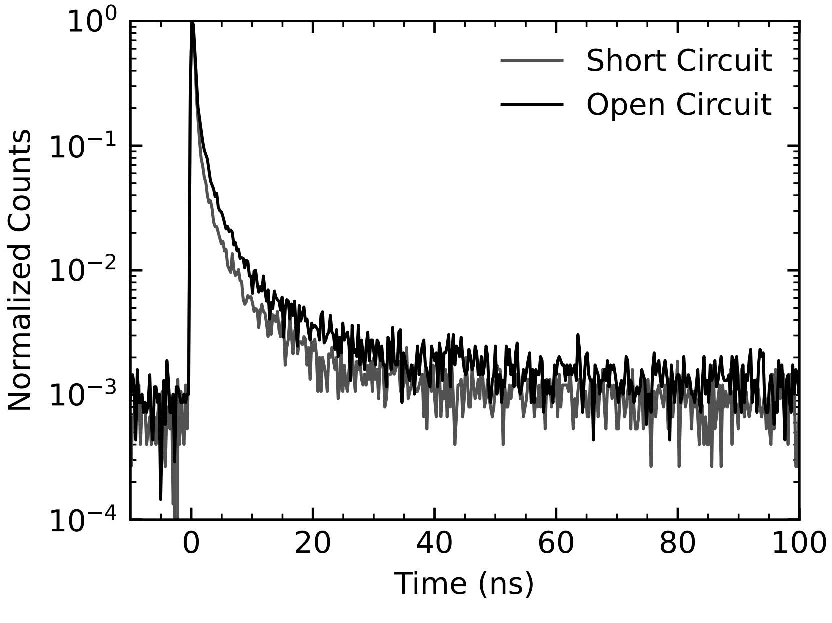

Figure S3: Time-resolved photoluminescence measurement of Cs17Br15 device in open and short circuit conditions.

2 ECS supporting data and models

2.1 Lineal photoluminescence and photocurrent

As described in the previous section, demodulating at the single frequency recovers the photoluminescence due to the single pulse. We determine the total PL/PC as the sum of both contributions and plot the total PL/PC vs fluence, Fig. S4.

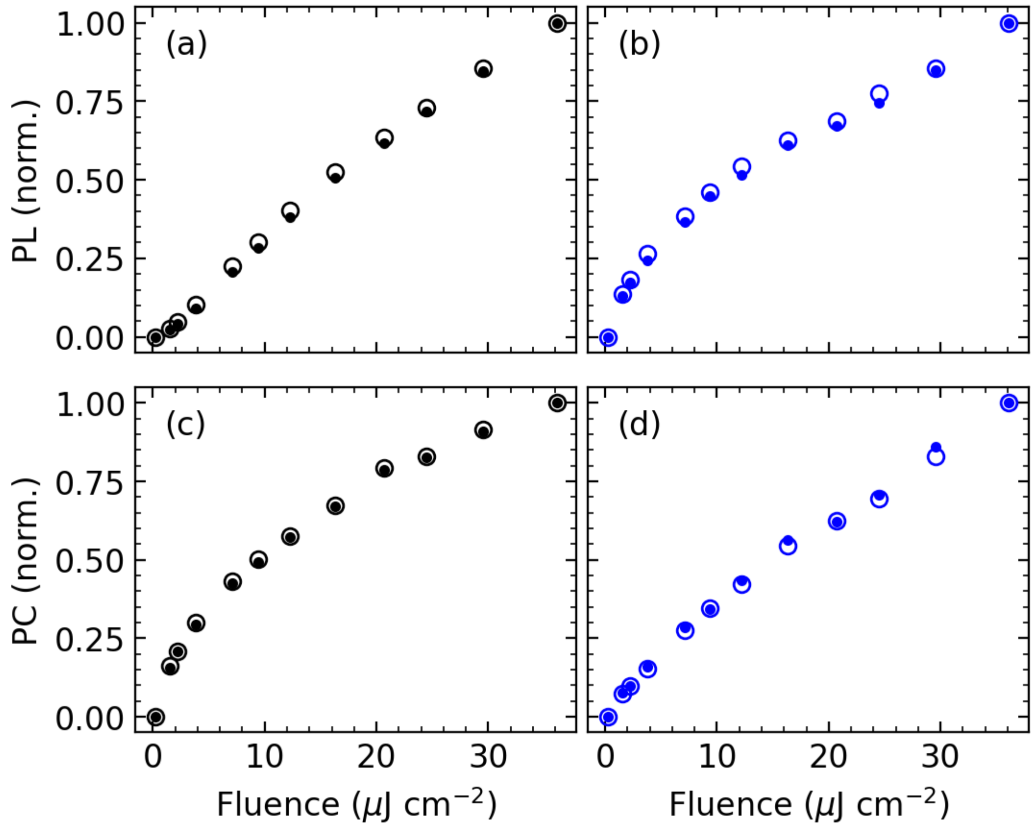

Figure S4: For Cs17Br15 fluence dependent single pulse (a) photoluminescence and (c) photocurrent. ITIC4F fluence dependent single pulse (b) photoluminescence and (d) photocurrent. Open marker corresponds to $t = 0$ ps and close marker corresponds to $t = 700$ ps.

2.2 Analytical models

2.2.1 Trap-assisted recombination:

ECPC:

$$n(t) = n(0)\exp(-\gamma_t N_t t) \quad \text{and} \quad p(t) = p(0)\exp(-\gamma_r n_t t). \tag{S17}$$

$$PC_{total} \propto \int_0^\tau n_1(t) + p_1(t)dt + \int_\tau^\infty n_2(t') + p_2(t')dt'. \tag{S18}$$

$$\begin{aligned} PC_{total} \propto & \int_0^\tau n(0)\exp(-\gamma_t N_t t) + p(0)\exp(-\gamma_r n_t t)dt \\ & + \int_\tau^\infty (n(\tau) + n(0))\exp(-\gamma_t N_t t') + (p(\tau) + p(0))\exp(-\gamma_r n_t t')dt'. \end{aligned} \tag{S19}$$

This is the same as

$$PC_{total} \propto 2n_0^2 + 2p_0^2 \tag{S20}$$

2.2.2 Bimolecular recombination:

If $Bnp >> \gamma_t N_t n$ equations 1, 2 and 3 from the main text simplify to:

$$\frac{dn}{dt} = G(t) - Bn^2 \tag{S21}$$

$$n = \frac{n_0}{1 + Bn_0 t} \quad \text{and} \quad n^2 = \frac{n_0^2}{(1 + Bn_0 t)^2} \tag{S22}$$

$$PL_{total} \propto \int_0^{\tau} n_1^2(t)dt + \int_{\tau}^{\infty} n_2^2(t-\tau)dt \tag{S23}$$

$$= \frac{n_0}{B}(1 - n(\tau)) + \frac{n_0}{B}(1 + n(\tau)) = 2n_0 \tag{S24}$$

The total photoluminescence corresponds to the contribution of the individual pulses. The expressions diverge in the case of photocurrent detection.

2.2.3 Auger recombination:

ECPL. Considering $\tau = 0$ still can be understood analytically.

$$\frac{dn}{dt} = -\gamma n - An^3. \tag{S25}$$

$$n(t) = \sqrt{\frac{\gamma/A}{(1 + \gamma/n_0^2 A)e^{2\gamma t} - 1}}. \tag{S26}$$

Integrated PL:

$$PL(n_0) \propto \int_0^{\infty} \frac{1}{e^2\gamma t - (1 + \gamma/n_0^2 A)^{-1}} dt \propto \left(1 + \frac{\gamma}{n_0^2 A}\right) \ln\left(\frac{\gamma + n_0^2 A}{\gamma}\right) \tag{S27}$$

The nonlinear PL

$$PL_{nl} = PL(2n_0) - 2PL(n_0) \tag{S28}$$

In the case where Auger recombination dominates $\frac{\gamma}{n_0^2 A} \to 0$ then:

$$PL_{nl} = \ln\left(\frac{\gamma + 4n_0^2 A}{\gamma}\right) - 2\ln\left(\frac{\gamma + n_0^2 A}{\gamma}\right) \tag{S29}$$

$$= \ln\left(\frac{\gamma^2 + 4n_0^2 A\gamma}{\gamma^2 + 2n_0^2 A\gamma + n_0^4 A^2}\right) \tag{S30}$$

The previous expression is negative if $\frac{\gamma}{n_0^2 A} < 1/2$, which is true based on our previous assumptions.

ECPC. We simplify to a steady state case due to the complex showing the sign of the signal. $\gamma n << An^3$. Then the nonlinear signal is clearly negative.

$$\frac{dn}{dt} = 0 = G - An^3 \tag{S31}$$

$$PC_{nl} = \frac{\left(2^{1/3} - 2\right) G^{1/3}}{A} \tag{S32}$$

2.3 Fitting procedures

2.3.1 Quasi-Steady

Solving equation 14 one obtains:

$$n(t) = \frac{n_0 \gamma/\beta}{(n_0 + \gamma/\beta)\exp(\gamma t) - n_0} \tag{S33}$$

Since we excite with a single pulse then the total photoluminescence measure is just the integral of the expression above, where R groups the photodiode response and the sample radiative response.

$$I_{PL} = R \int_0^{\infty} \frac{n_0 \gamma/\beta}{(n_0 + \gamma/\beta)\exp(\gamma t) - n_0} = \frac{1}{\beta}\ln(1 + n_0\beta/\gamma) \tag{S34}$$

By measuring a fluence dependence of the I_{PL}/n_0 we can extract the ratio β/γ and from the previously determined γ we isolated the β.

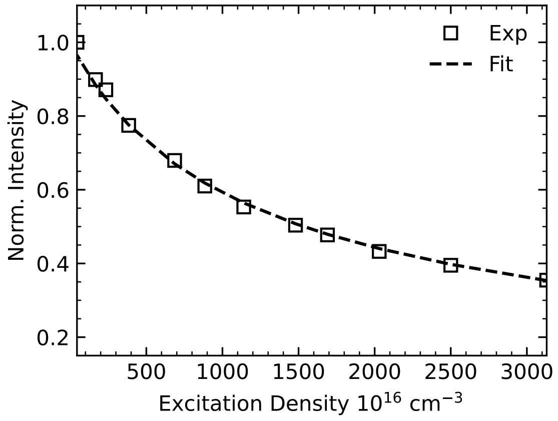

Figure S5: Normalized relative photoluminescence quantum yield as a function of the excitation density.

2.3.2 ECPL

The fits using Equation 14 are displayed in Figure S6. The measurement at fluence 1.0 μJ/cm^2 is shown here for demonstration purposes but the fit parameters are ignored due to the signal being dominated by noise.

2.3.3 ECPC

We fit only the right arm of the respective time traces. We consider the three lowest fluences a single or a double rising exponential, which is sufficient to describe the dynamics. We assign no physical value to the double exponential other than to

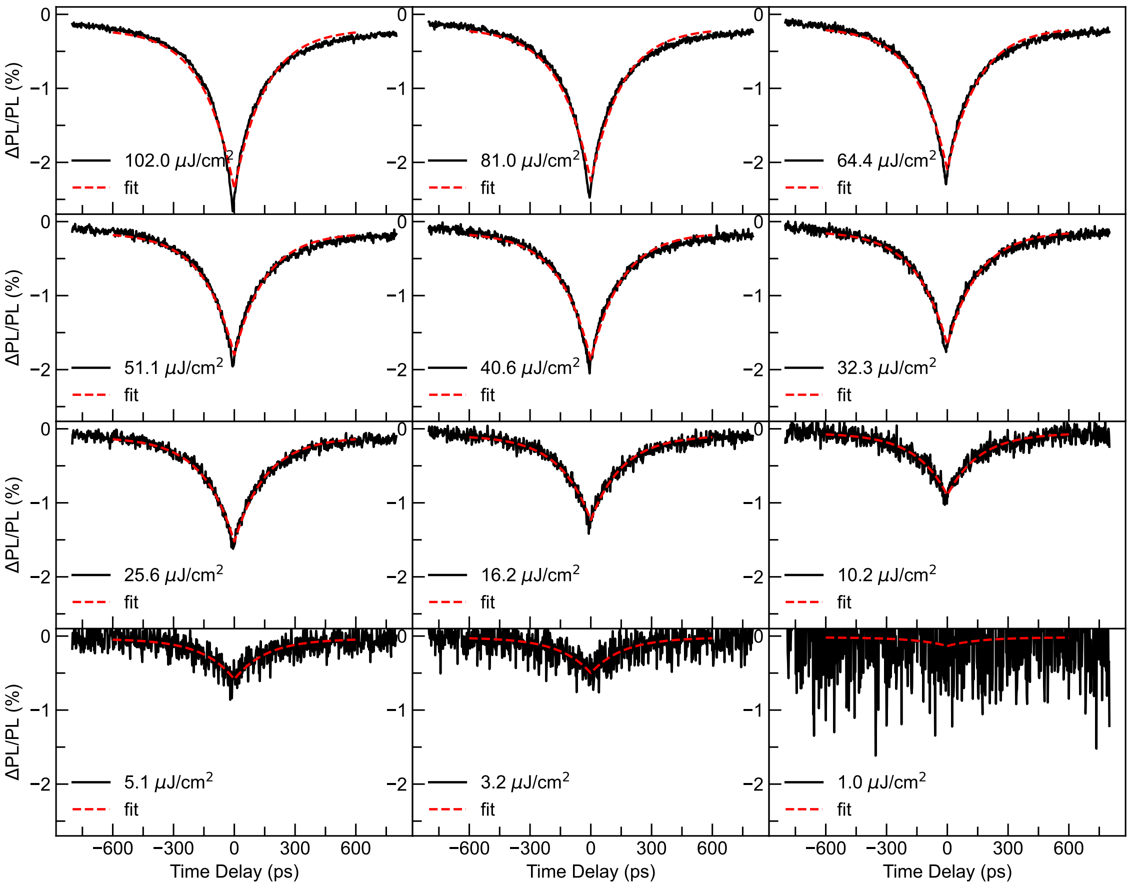

Figure S6: Fits (red dashed line) using equation 14 in the main article.

report an average rise time. As the fluence increases a decay in the signal can be observed and then we incorporate a single exponential. A general expression is shown in equation S35.

$$f(t) = A(1 - B\exp(-t/\tau_{r1}) - C\exp(-t/\tau_{r2})) + D\exp(-t/\tau_d) \qquad \text{(S35)}$$

We also note that in figure 3.b, at the highest fluences, the decay is very small, and therefore the estimation of the lifetime is not reliable. Instead, we focus on analyzing the rise time constants. The results of the fits are summarized in table 1

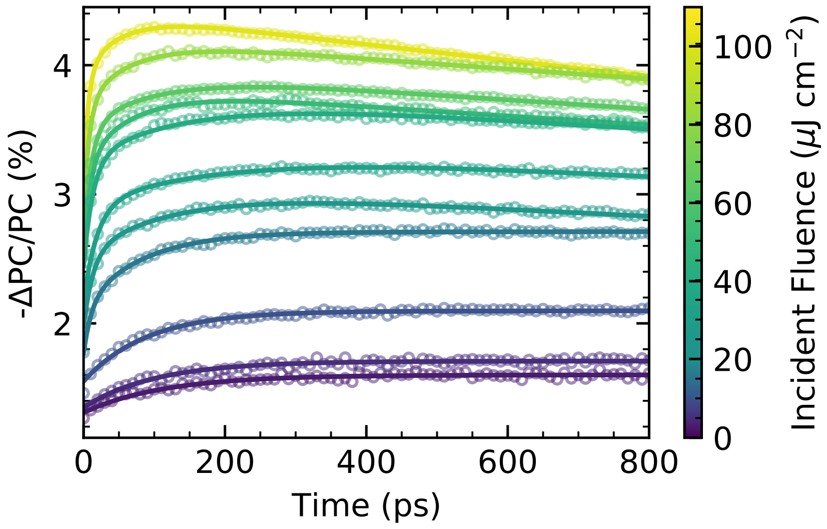

Figure S7: Fitting of the ECPC response of ITIC-4F, as describe in the text above.

Table 1: Summary of the extracted photophysical parameters for ITIC-4F from the ECPC measurements (figure 2.b of the main text).

Fluence (μJ cm^{-2})	τ_{r1} (ps)	τ_{r2} (ps)	τ_{avg} (ps)
3	113 $\pm$ 4	-	113 $\pm$ 4
5	102 $\pm$ 4	-	102 $\pm$ 4
10	91 $\pm$ 2	-	91 $\pm$ 2
16	84 $\pm$ 2	11 $\pm$ 2	56 $\pm$ 2
25	120 $\pm$ 4	13 $\pm$ 2	66 $\pm$ 4
32	196 $\pm$ 10	18 $\pm$ 4	89 $\pm$ 10
40	138 $\pm$ 5	13 $\pm$ 1	62 $\pm$ 5
51	70 $\pm$ 4	9 $\pm$ 2	37 $\pm$ 4
64	97 $\pm$ 5	11 $\pm$ 1	40 $\pm$ 5
81	66 $\pm$ 5	8 $\pm$ 2	30 $\pm$ 5
102	11 $\pm$ 5	6 $\pm$ 2	22 $\pm$ 5
